# Pseudoprogression after advanced first-line endocrine therapy in metastatic breast cancer with bone metastasis: A case report

**DOI:** 10.3389/fonc.2022.1099164

**Published:** 2023-01-04

**Authors:** Aijuan Tian, Huiyun Lv, Wei Liu, Jinbo Zhao, Shanshan Zhao, Kainan Wang, Chen Song

**Affiliations:** ^1^ Department of Nuclear Medicine, The Second Hospital of Dalian Medical University, Dalian, China; ^2^ Department of Oncology, The Second Hospital of Dalian Medical University, Dalian, China; ^3^ Department of Radiology, The Second Hospital of Dalian Medical University, Dalian, China

**Keywords:** pseudoprogression, flare, efficacy evaluation, bone metastasis, breast cancer

## Abstract

Approximately 75% of patients with advanced breast cancer develop bone metastasis, which significantly affects both the quality of life and the survival rate of patients. Accurate determination of the status of bone metastases is important for developing treatment strategies and the prognosis of the disease. Here, we report the case of a 33-year-old patient with advanced metastatic breast cancer (MBC) and multiple bone metastases, in which advanced first-line endocrine therapy and second-line chemotherapy were both considered unsuccessful according to the efficacy evaluation by conventional imaging. Considering the possibility of bone pseudoprogression, the original endocrine scheme was reapplied, and bone metastases achieved a great response of non-complete response (CR)/non-progressive disease (PD). This case showed that, in the course of therapy for the disease, if bone scintigraphy (BS) shows increased lesion density or new lesions, this probably indicates a favorable response (osteoblastic repair of osteolytic lesions) to therapy, and not the worsening of metastatic lesions, called bone pseudoprogression. This paper will provide new insights into strategies for the treatment of bone metastasis and shows the significance of distinguishing osteoblastic bone repair from real bone lesion progression in clinical settings.

## Introduction

Although the 5-year survival rate for early-stage breast cancer is around 80%, recurrence and metastasis nevertheless occur in 30%–40% of cases (1). Approximately 65%–75% of patients with metastatic breast cancer (MBC) develop bone metastasis. Bone is also the first site of metastasis for 27%–50% of patients with MBC (2). Skeletal complications of bone metastasis include bone pain, hypercalcemia, pathologic fractures, and spinal cord compression, all of which can greatly impair quality of life (3). However, although breast cancer with bone metastasis remains a virtually incurable disease, eliminating complications can improve quality of life and lead to better overall survival (OS). Standard treatments for bone metastasis are anticancer agents, such as chemotherapy and endocrine therapy, radiotherapy, and surgery. Bisphosphonates are generally used to prevent skeleton-related events.

Response to bone metastasis treatment is considered “unmeasurable” and is periodically estimated by using a combination of methods, including multiple kinds of imaging examinations, measurement of serum biochemical markers, and evaluation of patients’ symptoms (4, 5). Efficacy evaluation by imaging techniques is an essential part of the management of bone metastasis in breast cancer and is significant in the formulation of treatment plans and the clinical prognosis of patients. Imaging by single-photon emission computed tomography/computed tomography (SPECT/CT), computed tomography (CT), or magnetic resonance imaging (MRI) is a conventional part of evaluating bone metastases. CT scans, especially bone window scans, play a significant role in the evaluation of bone metastases response (6), and are superior to SPECT and MRI for showing clearly any changes in bone structure. Whole-body bone scans (WBSs) may identify metastases at an earlier stage and provide more information than radiography, CT, or MRI. Fluorodeoxyglucose F 18 ([ ^18^F]FDG) positron emission tomography/computed tomography( ^18^F-FDG PET/CT) has potential advantages over anatomical imaging in displaying changes in metabolic activity. By organically combining the functional phenomena of PET and the anatomical imaging of CT, it can show changes in metabolic activity before and after treatment for bone metastases, and it is more sensitive and specific than conventional imaging in detecting and evaluating bone metastases. The efficacy evaluation of bone metastases is of great importance in determining the appropriate treatment plan and clinical prognosis of patients. Although the diagnosis and treatment of bone metastases have been comprehensively improved, the efficacy evaluation of bone metastases is still less clear and controversial, and no consensus has been reached on the optimal imaging modality for this purpose. There is as yet no fully recognized standard for efficacy evaluation of bone metastases in breast cancer. Consistent, reproducible, and validated methods of assessing response to therapy have become even more important (7).

Fluorodeoxyglucose F 18 ([ ^18^F]FDG) is the most popular agent in tumor imaging and [ ^18^F]FDG PET/CT has become routine in clinical examination in recent years. It has played an important role in diagnosis, evaluation of tumor stage, and evaluation of efficacy. In terms of efficacy in bone metastases in breast cancer, it was found that some lesions with abnormally increased bone density had significantly lower glucose metabolism rates than osteolytic lesions and mixed lesions, demonstrating that local tumor cell proliferation is not actually active (8). This suggests that the enlargement or increase of osteogenic lesions indicated by CT or SPECT may be responsible for osteoblast repair rather than lesion progression, known as bone pseudoprogression.

The comprehensive use of various imaging methods to correctly determine the pseudoprogression of bone is important in the evaluation of the efficacy of bone metastasis in breast cancer; a diagnostic error may lead to a premature change in systemic drug scheme in clinics, which not only affects the choice of treatment plan and OS rate of patients but also shortens the application period of effective drugs. Herein, the case is reported of a premenopausal woman with advanced breast cancer with bone pseudoprogression that appeared after first-line therapy by CDK4/6 (cyclin-dependent kinase 4/6) inhibitors with aromatase inhibitors. We described the process of diagnosis, therapy, and efficacy assessment of skeletal lesions in detail, which should inform future clinical work.

## Case report

In March 2020, a 33-year-old woman was admitted to the Second Hospital of Dalian Medical University with the principal complaint of having a painless lump in her right breast, which she found accidentally. The breast ultrasonography showed a 2.8 cm × 2.7 cm × 1.2 cm mass in the right breast, which was classified as 4C by the breast imaging reporting and data system (BI-RADS). A core needle biopsy was performed on 23 March 2020, and the subsequent pathology revealed adenocarcinoma (from the punctured tissue of the right breast mass)—non-specific invasive breast cancer grade 2—part of which was a high-nuclear-grade ductal carcinoma *in situ.* The immunohistochemical (IHC) report revealed ER (70%+), PR (50%+), HER-2 (1+), Ki-67 (about 30%+) and BRCA1 (−). At the same time, the CT scan showed no obvious abnormalities in the liver, brain, or lung; however, WBS suggested the possibility of bone involvement in malignant lesions. To be more accurate and for comprehensive staging, the patient underwent [ ^18^F]FDG PET/CT examination on 31 March 2020, which showed multiple lymph nodes metastases (in the right axilla) and bone metastases (in the anterior coracoid process of the right scapula, 1st thoracic vertebra, first, second and fourth lumbar vertebrae, and right ilium) (**Supplementary Figure 1**). All bone metastases showed osteolysis and increased glucose metabolism, suggesting that tumor cells proliferated actively at the lesion. The patient had no family history or genetic history of cancer. She was in good health and had no medical history of hypertension or diabetes or smoking, drinking, or other bad habits. In the end, the patient was diagnosed with grade 2 invasive breast cancer with lymph node metastasis in the right axillary and bone metastasis, cT2N3M1, stage IV.

**Figure 1:**
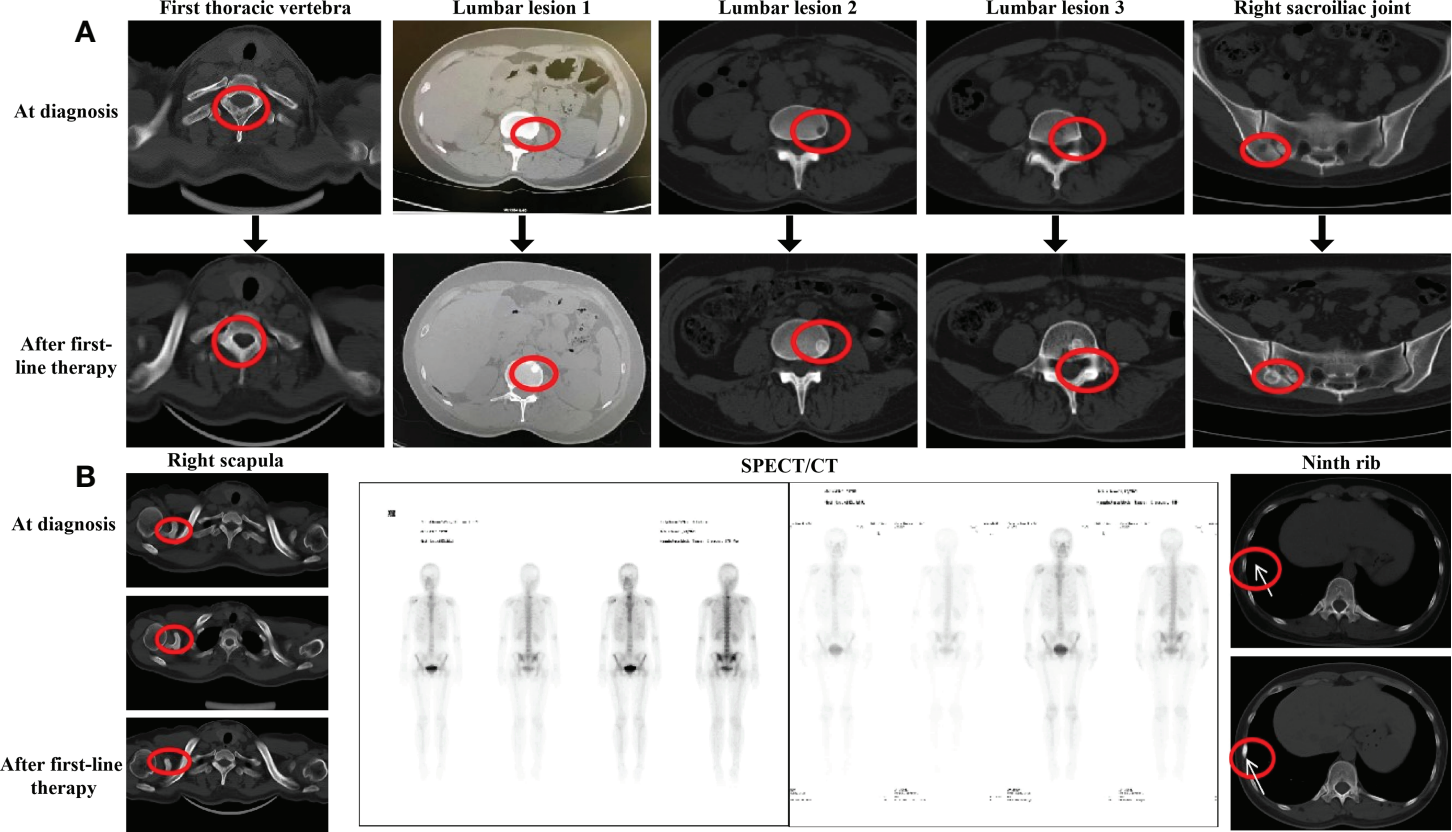
Evaluation after first-line endocrine therapy **(A)** CT evaluation of the bons lesion after first-line endocrine therapy in September 2020. **(B)** CT evaluation of right scapula the ninth rib and SPECT evaluation after first-line endocrine therapy in September 2020.

The patient was administered advanced first-line endocrine therapy with palbociclib [125 mg po (per os, orally) qd (quaque die, daily) d1–d21, q28d] combined with exemestane (25 mg po qd) for six cycles from 30 March to 16 September 2020. During the same period, goserelin was used to suppress ovarian function and ibandronate monosodium was used to treat bone metastasis. During endocrine therapy, laboratory findings showed levels of tumor markers, and related biochemical indicators showed a slight decrease or a stable trend. The patient underwent CT and WBS on 16 September 2020 to assess efficacy. The CT showed that the primary lesion of the right breast reduced (59%), shrinking to 1.3 cm × 0.8 cm. WBS showed that the bone metabolism of the lumbar vertebra, right sacroiliac joint, and first thoracic vertebra decreased or even disappeared with increased bone density on CT (**Figure 1A**). The bone metabolism of the right scapula was similar, but its bone density on a CT scan showed an increase first and then a decline, suggesting that bone metastasis was progressing (**Figure 1B**, left). In addition, new nuclide-concentrated foci appeared in the right ninth rib, as shown by WBS, and the bone density of the lesion increased after endocrine therapy, so this was identified as a new lesion (**Figure 1B**, center and right). Based on the examination results at that time for the bone metastases and efficacy, the disease was categorized tentatively as progressive disease (PD). This meant that first-line endocrine therapy was not effective and the treatment plan should be changed.

**Figure 2:**
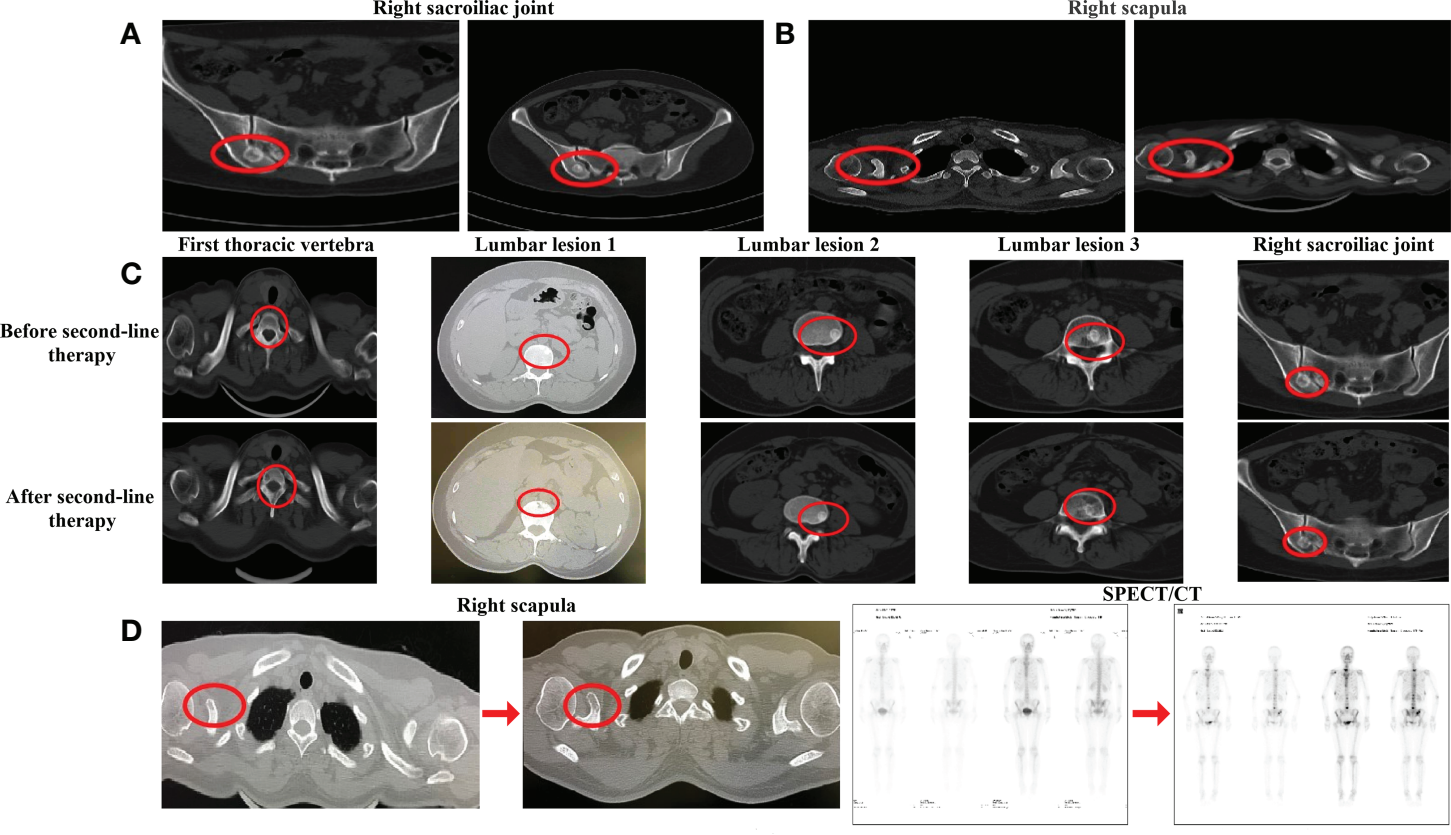
Evaluation after second-line chemotherapy. **(A)** CT evaluation of the right sacroiliac joint before chemotherapy and after cycles in December 2020. **(B)** CT evaluation of the right scapula before chemotherapy and after chemotherapy in May 2021 **(C)** CT evaluation of the bone lesion after second-line therapy in July 2021, **(D)** CT evaluation of the right scapula and SPECT evaluation after second-line therapy in July 2021.

From 22 September 2020, albumin-bound paclitaxel [200 mg intravenous glucose tolerance test (IVGTT) d1, d8, q21d] combined with capecitabine [1,500 mg po qd d1–d14, q21d] was administered as the second-line chemotherapy for six cycles, with ibandronate monosodium continuing. After four cycles of combined therapy, the CT scan showed that, despite most of the bone metastases being similar to before, the edge of the right iliac lesion had begun to blur (**Figure 2A**). Moreover, the level of alkaline phosphatase (ALP) and lactate dehydrogenase (LDH) as well as CA125 had increased at the end of third cycle. All of these indicated the progression of the disease, and that the current treatment efficacy was poor. We switched to maintenance treatment with capecitabine alone for eight cycles and the patient continued to receive ibandronate monosodium injections every month. On 6 May 2021, the CT examination after the first two cycles of capecitabine alone showed different degrees of osteolytic changes in the right scapula (**Figure 2B**). Because the patient complained that the oral painkillers contributed nothing to the pain relief, overloaded doses of ibandronate monosodium were administered. At the end of second-line chemotherapy, an examination with CT and SPECT/CT scans was conducted on 26 July 2021, which indicated that the skeletal lesion had progressed again. The size of the right breast tumor continued to reduce; however, in comparison with the CT and SPECT/CT images before chemotherapy, most metastatic bone lesions showed osteolytic changes. The new osteolytic lesions were also found in the thoracic vertebra, the first and third lumbar vertebrae, and the right sacroiliac joint (**Figure 2C**), and the original osteolytic lesion in the coracoid process of the right scapula was enlarged (**Figure 2D**, left). In addition, WBS demonstrated that there were multiple new nuclide-concentrated foci. Combined with the decreased density of bone lesions, increased tumor markers, and the exacerbation of bone pain symptoms, after efficacy evaluation the disease was categorized as PD again (**Figure 2D**, right); the advanced second-line chemotherapy was declared a failure.

**Figure 3:**
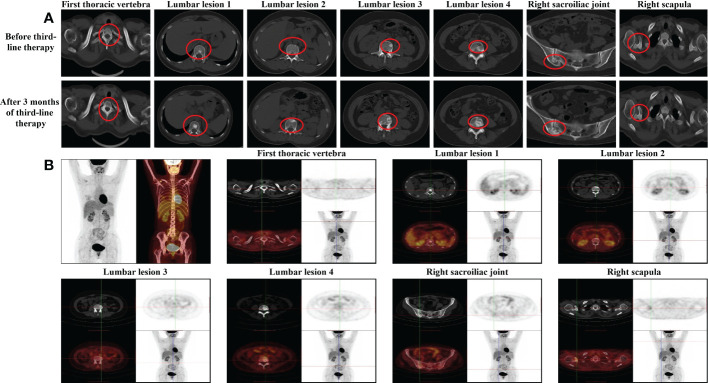
Imaging examinations during the third-line endocrine therapy **(A)** CT evaluation of the bone lesion after 3 months of third-line endocrme therapy in October 2021. **(B)** PET/CT evaluation of each bone lesion after 3 months of third-line endocrine therapy in October 2021.

With the failure of advanced first-line and second-line therapy, we reviewed the patient’s previous imaging outputs and found that the changes in bone lesions after first-line endocrine therapy may be similar to bone pseudoprogression and considered that the first-line efficacy evaluation may be wrong. Despite the lack of evidence-based medical evidence and guidelines, after multidisciplinary treatment (MDT), we changed the treatment regimen to the original endocrine regimen. During 3 months of the re-administration, the patient’s clinical manifestations and other indicators generally improved, the CT scan revealed that the scope of osteogenic lesions expanded, and the density increased (**Figure 3A**). In October 2021, approximately three cycles after endocrine therapy administration, the patient received radiotherapy as a synergistic treatment. [ ^18^F]FDG PET/CT in the same month demonstrated that the FDG uptake in multiple original bone lesions (thoracic vertebrae, lumbar vertebrae, and right sacroiliac joints) decreased significantly or disappeared, and lesion density changed from osteolysis into osteogenesis (**Figure 3B**). All the above conditions were considered to be a sign of reactive osteogenesis after treatment for the metastatic tumor. Because of the results of [ ^18^F]FDG PET/CT scans and the improvement of clinical manifestations, we believed that tumor proliferation of bone metastases was inhibited. The increased osteogenesis phenomenon was considered to be osteoblastic repair; this suggested that the current treatment options were effective.

**Figure 4:**
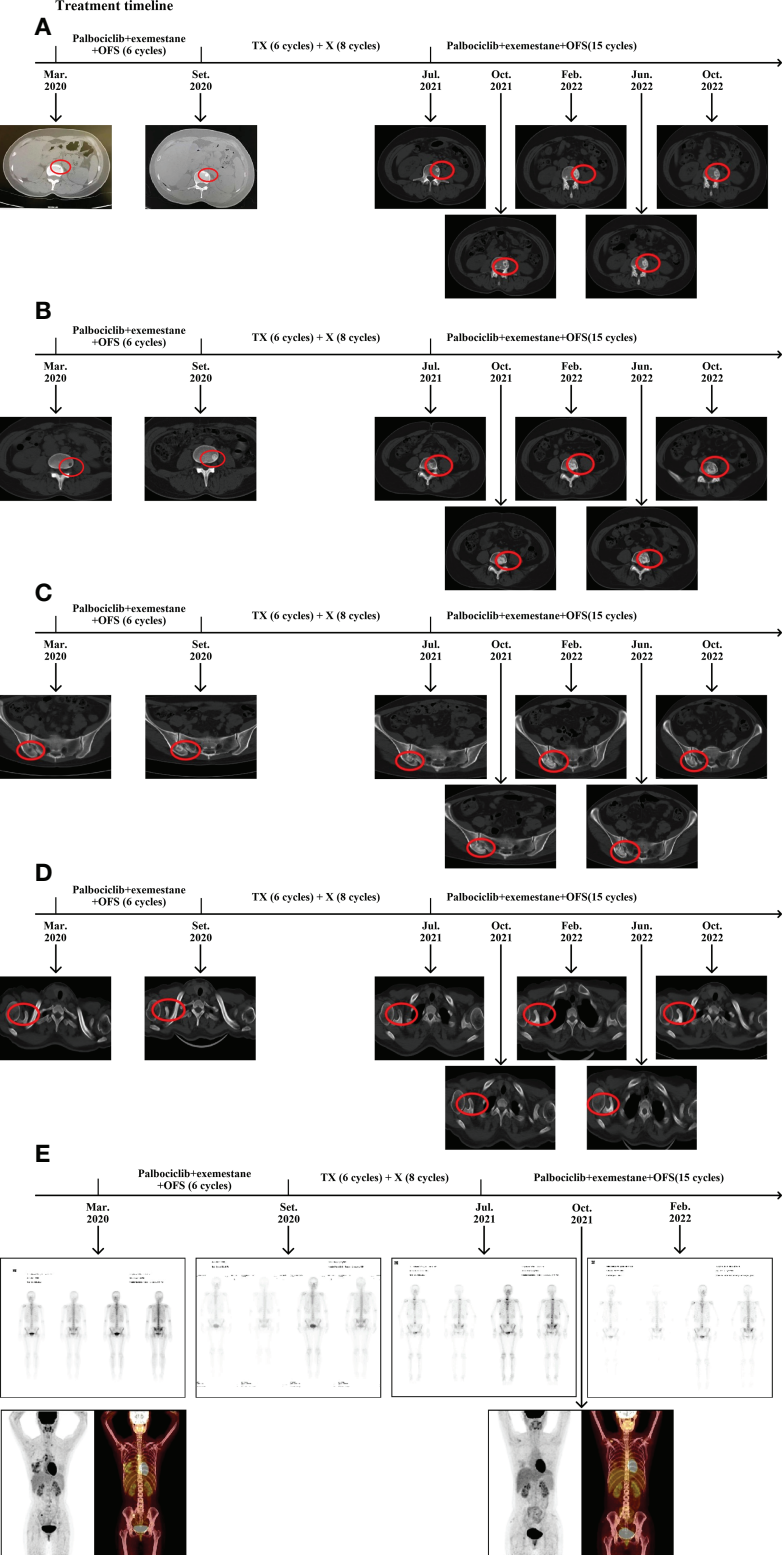
Changes in bone lesions during the treatment. **(A)** CT evaluation of the lumbar lesion 1 during the treatment. **(B)** CT evaluation of lumbar the lesion 2 during the treatment. **(C)** CT evalution of the right sacroiliac joint during the treatment. **(D)** CT evaluation of the coracoid process of the right scapula during the treatment. **(E)** SPECT/CT evaluation during the treatment. T: Albumin paclitaxel; X: Albumin paclitaxel.

After seven cycles, a breast CT scan showed a significant reduction in the lesion size in the right breast (the maximum measurement diameter was about 0.6 cm); and after 15 cycles (October 2022) the lesion size remained stable. After 10 cycles, the osteoblastic range of each bone metastasis lesion continued to increase, and osteolytic lesions of the right scapular acoid began to show osteogenic changes (**Figures 4A–D**). SPECT/CT scan indicated increased bone density and a decrease in the number of bone-concentrated foci and the degree of nuclide concentration (**Figure 4E**). Those changes were considered for reactive osteogenesis after multiple bone metastases therapy. The results of relevant laboratory findings are also very important and support the efficacy evaluation. We systematically reviewed tumor markers such as CEA, CA153, and CA125 during the whole treatment, which were all within the normal range, although there were changes during different therapy stages. The values of ALP and LDH showed obvious variation at different stages of the treatment. The levels of both enzymes fluctuated within the normal range during endocrine therapy, both increased to varying degrees during the advanced second-line chemotherapy, and both decreased to within the normal range after the original endocrine therapy was applied (**Supplementary Figures 2**, **3**). In addition, the latest treatment relieved pain and improved the patient’s health-related quality of life. Above all, the efficacy evaluation achieved a great response of non-CR/non-PD, and the original endocrine therapy was a hard-won success. At the time of writing, the patient had been receiving endocrine therapy for 15 months and remained progression free, showing good tolerability and a high quality of life.

## Discussion

As far as breast cancer is concerned, the incidence of bone metastasis is high, patient quality of life is poor, and there are many changes after therapy. Diagnosis, treatment, and efficacy evaluation are challenges that need to be addressed in clinical practice. Internationally, there are four main criteria for the efficacy evaluation in bone metastases: those of the International Union Against Cancer (UICC) (9), the World Health Organization (WHO) (10), the MD Anderson Cancer Center USA (MDA) (2), and the Positron Emission Tomography Response Criteria in Solid Tumor (PERCIST) (11). The WHO’s efficacy evaluation criteria for bone metastases of 1981 declares that partial response (PR) includes “decreased density of blastic lesions for at least four weeks”, and progressive disease (PD) is defined as an “increase in the size of existent lesions or appearance of new lesions” (10), which is not universally accepted by clinical experts around the world because it is not consistent with clinical cases.

In 2018, a Chinese professor, Song Santai, proposed that the increase and enlargement of osteoblastic lesions should not be understood to automatically indicate the progression of bone metastasis and may instead be a sign of effective treatment in certain circumstances. Decreased bone density and osteolytic changes that occurred in osteoblastic lesions were the symptoms of deterioration when the next-line therapeutic scheme should be initiated (12). Although in complete contradiction with the WHO’s guidance, Song’s view has already been verified in clinical settings successfully, and more and more clinicians around the world raise doubts about the WHO’s criteria. In recent years, new bony lesions that may represent osteoblastic bone healing have been studied extensively and defined as bone pseudoprogression. In 2021, Professor Zhang Jian and his team launched a clinical trial that used WBS to monitor disease progression in bone in 48 patients with hormone receptor-positive MBC. It was found that osteoblastic new bony lesions detected on follow-up may represent bone pseudoprogression (13). Huang et al. reported that a woman with MBC had pseudoprogression after first-line therapy that included palbociclib combined with exemestane (14). At the time of writing, all published articles about bone pseudoprogression in breast cancer have involved HR-positive patients who developed bone pseudoprogression during or after endocrine therapy. However, no studies have proved a direct connection between bone pseudoprogression and endocrine therapy. A large number of scholars attribute this to the fact that HR-positive patients account for the largest proportion of breast cancer patients, and bone metastasis is a common occurrence in MBC.

The presence of metastatic lesions in breast cancer can influence bone homeostasis to favor bone resorption or bone formation by affecting the activity of osteoclasts or osteoblasts, thereby resulting in osteolytic, osteoblastic, or mixed lesions (15, 16). It is known that most bone metastases in breast cancer are osteolytic (17). Although osteoblastic metastases in breast cancer are relatively rare, it is easy to misdiagnose and initiate the wrong treatment. It should be noted that not all newly emerging skeletal lesions, increases in skeletal lesion density, and expanded ranges of skeletal lesions are indicative of progression; this may be osteogenic repair after treatment of osteolytic lesions and a manifestation of effective therapy (13, 14). Therefore, we need to explore the combination of multiple imaging methods to accurately evaluate the response of bone metastases to treatment. Effective treatment should be continued if patients’ clinical manifestations are relieved, and osteogenesis is observed.

In this case, the efficacy evaluation of advanced first-line endocrine therapy was not completely correct. Reviewing the course of the disease, initial imaging seemed to indicate bone flare, and the increased and enlarged lesions after first-line endocrine therapy were mistaken for progression, which misled the clinical evaluation of endocrine resistance, thus a premature switch to chemotherapy that was harmful to the patient. Scintigraphic bone flare sign is characterized by an increase in the intensity of tracer uptake at the sites of bone metastases and/or the appearance of “new” lesions shortly after the commencement of treatment (18, 19). The phenomenon referred to as new or more prominent osteoblastic bony lesions arises in the tumor lesions because of effective therapy. Osteoblasts mediate bone healing, and an early increase in osteoblast activity following successful systemic therapy has been observed, as evidenced by increased radiotracer uptake on WBS. Some serial biochemical measurements of osteoblast function also confirmed the flare response (20). As a result, bone flares can be considered a sign of therapeutic efficacy. However, the osteolytic lesion that has been overlooked on WBS before therapy might also present a new site of radiotracer uptake. Therefore, the patient may be misinterpreted as indicating possible PD (21). In our case, all bone metastases were osteolytic lesions when they were diagnosed, and glucose metabolism of all lesions increased, suggesting tumor cells proliferate vigorously. After first-line endocrine therapy, a CT scan showed that the possibility of the osteoblastic bone repair of osteolytic lesions was considered. In addition, the re-examination of WBS revealed that a new lesion had appeared. The above situations suggest that, through effective treatment, not only does osteolytic bone destruction turn into osteoblastic bone repair, but those tiny osteolytic lesions that cannot be detected by conventional imaging also show osteogenic repair. As a result, both new lesions and enlarged lesions observed on later imaging were actually the results of osteoblastic bone repair, and the number of bone repair lesions after treatment is often greater than the number of original sites of osteolytic destruction (22).

Therefore, when the progression was defined after second-line chemotherapy, we re-analyzed images carefully and finally defined relevant evidence of increased bone lesion density on bone window CT during endocrine therapy through repeated comparison. There was a great possibility that the osteoblastic flare phenomenon had occurred; the progression during this period was considered to be bone pseudoprogression. The appearance of these lesions as a result of osteoblastic repair proved that the patient was sensitive to endocrine therapy, so the original endocrine scheme was resumed. This choice was based on an adequate analysis of the previous images and knowledge of pseudoprogression, although it was without support from evidence-based medicine and guidelines. During the treatment of the original endocrine scheme, results of periodic bone window CT scans demonstrated that all bone metastases had successively exhibited osteoblastic changes, and the osteoblastic range was continuously expanding. SPECT/CT tomographic fusion imaging also confirmed that increased bone density and decreased degree of concentration were osteoblastic repair changes after treatment. The efficacy of bone metastases was evaluated as non-CR/non-PD, and, combined with the reduction of the primary lesion, the improvement of clinical symptoms, and the decrease of tumor markers, the original endocrine therapy was considered effective.

There were still two limitations during the treatment. We did not perform a needle biopsy to confirm the pathological diagnosis of multiple bone metastases, and we did not incorporate the corresponding biochemical markers to evaluate efficacy. Needle biopsy is an invasive procedure; it is not ethical to perform a needle biopsy on every bone metastasis. Clinically, we usually make a judgment through imaging and other non-invasive methods. Besides, when local small lesions or a small number of lesions change, the corresponding biochemical markers often do not increase enough to show the change sensitively. At this time, the most effective method is to evaluate by imaging and patient symptoms, which also highlights the significance of imaging methods in evaluating the efficacy of bone metastases.

With immunotherapy becoming a more popular practice, pseudoprogression is a common phenomenon. The possibility of osteoblastic flare should be considered to avoid a misinterpretation of radiological findings, emphasizing that accurate efficacy evaluation of imaging plays a pivotal role throughout the treatment. Our case report points out that timely follow-up imaging and a critical analysis of both clinical and iconography evolution are vital for making the right therapeutic decisions (23). With the findings assessed by WBS, CT, SPECT/CT, and [ ^18^F]FDG PET/CT in this case, and in conjunction with other studies on the progression of pseudobone, it is not clear which imaging modality can be isolated to assess accurate response in bone metastasis. From our point of view, the best imaging modality to assess accurate response in bone metastasis is a combination of various imaging methods, and it is significant to compare the density change of the same bone metastasis site before and after treatment. When different imaging results are contradictory, [ ^18^F]FDG PET/CT is recommended to clarify the efficacy evaluation. In future clinical research, we will continue to work to build a diagnosis and treatment model for early detection and diagnosis of bone pseudoprogression to make progress in the study of bone metastasis pseudoprogression of breast cancer.

## Conclusion

This is the first clinical case of pseudoprogression in a patient who changed to the original endocrine therapy scheme after pseudoprogression was found. Although the imaging progression, the patient’s clinical manifestations improved during endocrine therapy. Clinicians should be aware of the possibility of bone pseudoprogression in an MBC patient with bone metastasis. We must analyze and observe the changes carefully, and pharmacotherapy should not be hastily discontinued. On the basis of improvement of clinical symptoms, we must analyze and observe the changes carefully, and should not change the treatment plan hastily.

## Data availability statement

The original contributions presented in the study are included in the article/**Supplementary Material**. Further inquiries can be directed to the corresponding author.

## Ethics statement

Written informed consent was obtained from the individual(s) for the publication of any potentially identifiable images or data included in this article.

## Author contributions

All authors made substantial contributions to conception and design, acquisition of data, or analysis and interpretation of data; took part in drafting the article or revising it critically for important intellectual content; gave final approval of the version to be published; and agree to be accountable for all aspects of the work.
