# Supplementary figures and images for: Pseudoprogression after advanced first-line endocrine therapy in metastatic breast cancer with bone metastasis: A case report
Source: Front Oncol. 2023 Jan 4;12:1099164. doi: 10.3389/fonc.2022.1099164 (PMC9845761; doi:10.3389/fonc.2022.1099164)

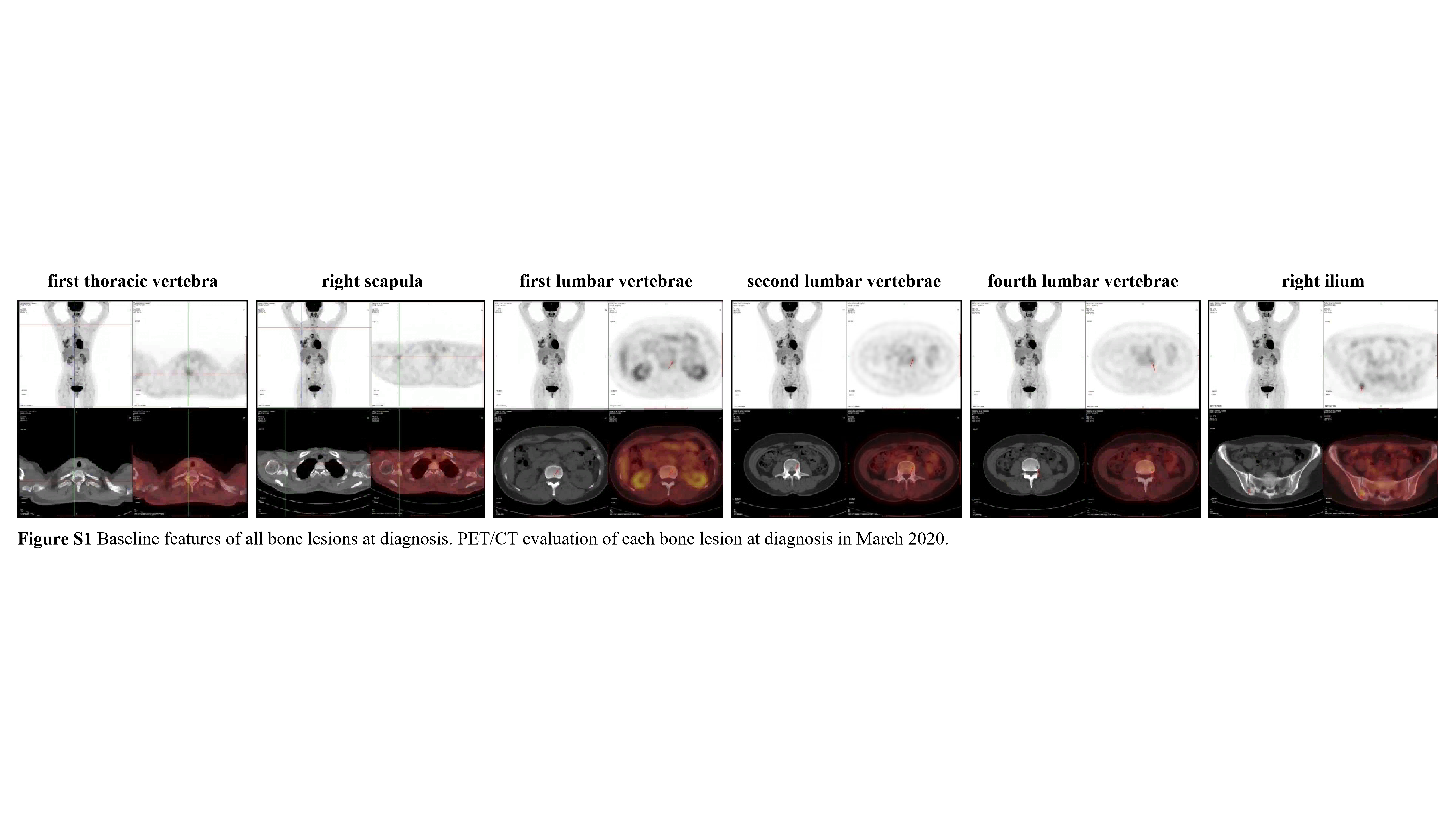

Supplement: Supplementary file 1 [file Image_1.tif]

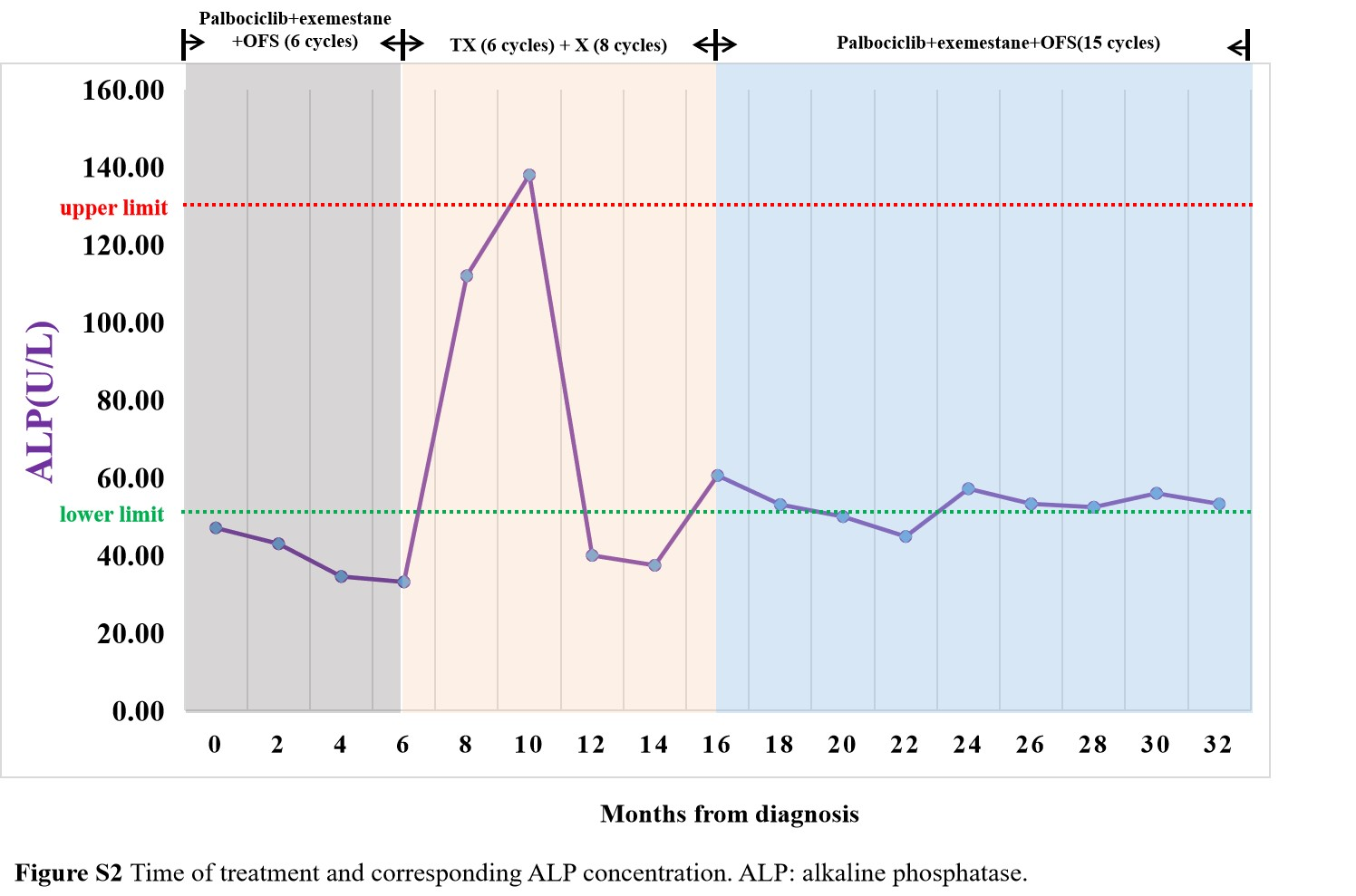

Supplement: Supplementary file 2 [file Image_2.tif]

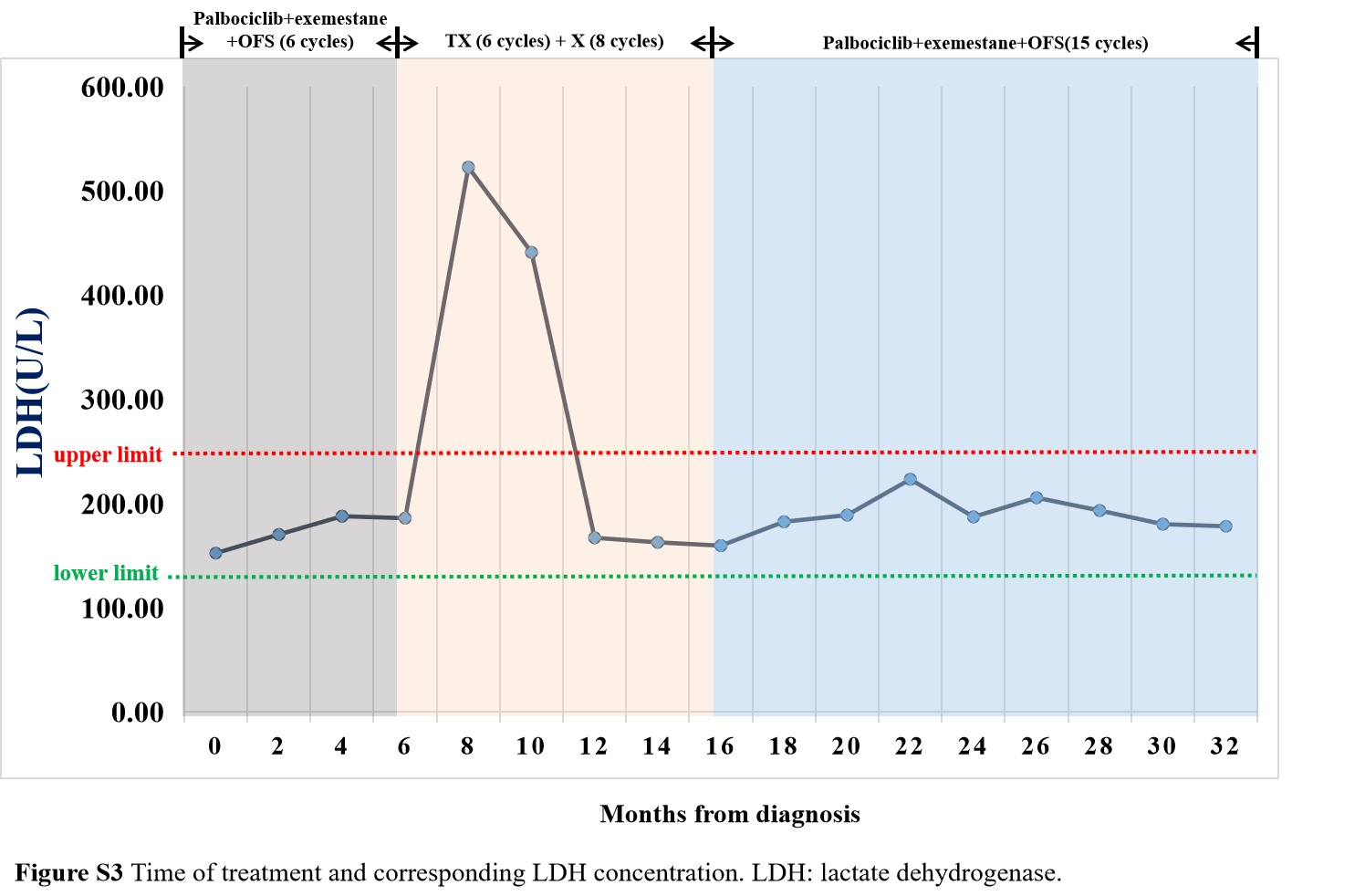

Supplement: Supplementary file 3 [file Image_3.tif]
